# Cardioprotection by poloxamer 188 is mediated through increased endothelial nitric oxide production

**DOI:** 10.1038/s41598-025-97079-z

**Published:** 2025-04-30

**Authors:** Gaoxian Chen, Hunter F. Douglas, Zhu Li, William J. Cleveland, Claudius Balzer, Demetris Yannopoulos, Ian Y. Chen, Detlef Obal, Matthias L. Riess

**Affiliations:** 1 Department of Anesthesiology, Perioperative, and Pain Medicine, Stanford University, Stanford, CA USA; 2 Department of Anesthesiology, TVHS VA Medical Center, Nashville, TN USA; 3 Department of Anesthesiology, Vanderbilt University Medical Center, Nashville, TN USA; 4 Department Pharmacology, Vanderbilt University, Nashville, TN USA; 5 Division of Cardiology, Department of Medicine, University of Minnesota, Minneapolis, MN USA; 6 Departments of Medicine (Cardiovascular Medicine) and Radiology, Stanford University School of Medicine, Stanford, CA USA; 7 Medical (Cardiology) and Radiology Services, Veterans Affairs Palo Alto Health Care System, Palo Alto, CA USA; 8 Stanford Cardiovascular Institute, Stanford University, Stanford, CA USA; 9 Department of Anesthesiology, University of Iowa, Iowa, IA USA

**Keywords:** Cardiac arrest, Copolymer, Ischemia/reperfusion injury, Myocardial infarction, NO, P188, Cardiovascular biology, Cell death, Pharmacology

## Abstract

Ischemia/reperfusion (I/R) injury significantly contributes to the morbidity and mortality associated with cardiac events. Poloxamer 188 (P188), a non-ionic triblock copolymer, has been proposed to mitigate I/R injury by stabilizing cell membranes. However, the underlying mechanisms remain incompletely understood, particularly concerning endothelial cell (EC) function and nitric oxide (NO) production. We employed human induced pluripotent stem cell (iPSC)-derived cardiomyocytes (CMs) and ECs to elucidate the effects of P188 on cellular survival, function, and NO secretion under simulated I/R conditions. iPSC-CMs contractility and iPSC-ECs’ NO production were assessed following exposure to P188. Further, an isolated heart model using Brown Norway rats subjected to I/R injury was utilized to evaluate the ex-vivo cardioprotective effects of P188, examining cardiac function and NO production, with and without the administration of a NO inhibitor. In iPSC-derived models, P188 significantly preserved CM contractile function and enhanced cell viability after hypoxia/reoxygenation. Remarkably, P188 treatment led to a pronounced increase in NO secretion in iPSC-ECs, a novel finding demonstrating endothelial protective effects beyond membrane stabilization. In the rat isolated heart model, administration of P188 during reperfusion notably improved cardiac function and reduced I/R injury markers. This cardioprotective effect was abrogated by NO inhibition, underscoring the pivotal role of NO. Additionally, a dose-dependent increase in NO production was observed in non-ischemic rat hearts treated with P188, further establishing the critical function of NO in P188 induced cardioprotection. In conclusion, our comprehensive study unveils a novel role of NO in mediating the protective effects of P188 against I/R injury. This mechanism is evident in both cellular models and intact rat hearts, highlighting the potential of P188 as a therapeutic agent against I/R injury. Our findings pave the way for further investigation into P188’s therapeutic mechanisms and its potential application in clinical settings to mitigate I/R-related cardiac dysfunction.

**Supplementary Information:**

The online version contains supplementary material available at 10.1038/s41598-025-97079-z.

## Introduction

More than 380,000 patients in the United States experience of out-of-hospital cardiac arrest each year^1^. Even with the most effective cardiopulmonary resuscitation (CPR), more than 90% of patients suffer serious neurological damage or die^2^. Although most of patients receive early first responder CPC in most metropolitan areas in the US^3^, followed by professional emergency medical services (EMS) arrival on average 8 to 12 min later, abrupt reintroduction of blood flow at the initiation of CPR after prolonged ischemia exacerbates tissue recovery and potentiates the resulting ischemia/reperfusion (I/R) injury^4^.

The intricate interplay between cellular membranes and pathological stress responses, particularly during I/R injury, has garnered considerable attention in the realm of biomedical research. Copolymer-based cell membrane stabilizers (CCMS), comprising hydrophobic poly-propylene oxide (PPO) and hydrophilic poly-ethylene oxide (PEO) chains, have emerged as potent agents in mitigating membrane damage and curtailing cell death pathways^5–8^. Among these, Poloxamer 188 (P188, E_75_P_30_E_75_, Sigma Aldrich), a non-ionic tri-block copolymer, has been spotlighted for its ability to bridge gaps in compromised cell membranes^9^, thereby obstructing the formation of detrimental membrane pores under conditions of intense cellular stress experienced during ischemia^8^. The protective mechanism of P188 extends beyond mere physical membrane repair; it encompasses the prevention of unregulated ion flux across cellular boundaries and the blockade of apoptosis by maintaining calcium equilibrium and preventing mitochondrial depolarization under oxidative stress^6,10^. This dual action not only safeguards skeletal muscle and neurons from I/R-induced damage but also fortifies the blood-brain barrier against various cerebral challenges, significantly reducing brain edema and neuronal cell death^11–17^. Both, in-vivo and ex-vivo models of cardiac I/R injury demonstrated the protective effect of P188 against cardiomyocyte (CM) cell necrosis and apoptosis when administered at the onset of reoxygenation^7^.

Building on the foundational role of the vascular endothelium in I/R injury, our research delves into the mechanisms by which endothelial cells (ECs) mediate a protective response against such injuries, specifically through the modulation of nitric oxide (NO) production^18–21^.

To test this hypothesis, we employed a multifaceted experimental design encompassing human induced pluripotent stem cell-derived CMs (iPSC-CMs) and iPSC-ECs, as well as rat isolated, intact hearts. iPSCs provide a robust platform for studying ischemia-reperfusion injury while overcoming the limitations of primary cardiomyocytes, such as limited availability, donor variability, and rapid dedifferentiation in vitro^22–25^. By evaluating P188’s influence on NO signaling in EC, we aimed to determine its impact on CM viability and cardiac function following hypoxia/reoxygenation. Our study focuses on endothelial-derived NO as a potential mediator of P188-induced cardioprotection, highlighting its ability to modulate endothelial function and oxidative stress pathways.

Through this research, we provide new insights in myocardial I/R injury, positioning NO not merely as a passive mediator but as a central player in the protective effects of P188. By elucidating the complex interplay between P188, endothelial NO production, and CM survival, this study provides a foundation for future therapeutic strategies aimed at mitigating I/R in the heart.

## Results

### iPSC-derived cell studies

#### iPSC-CM function after hypoxia/reoxygenation experiments

**Fig. 1:**
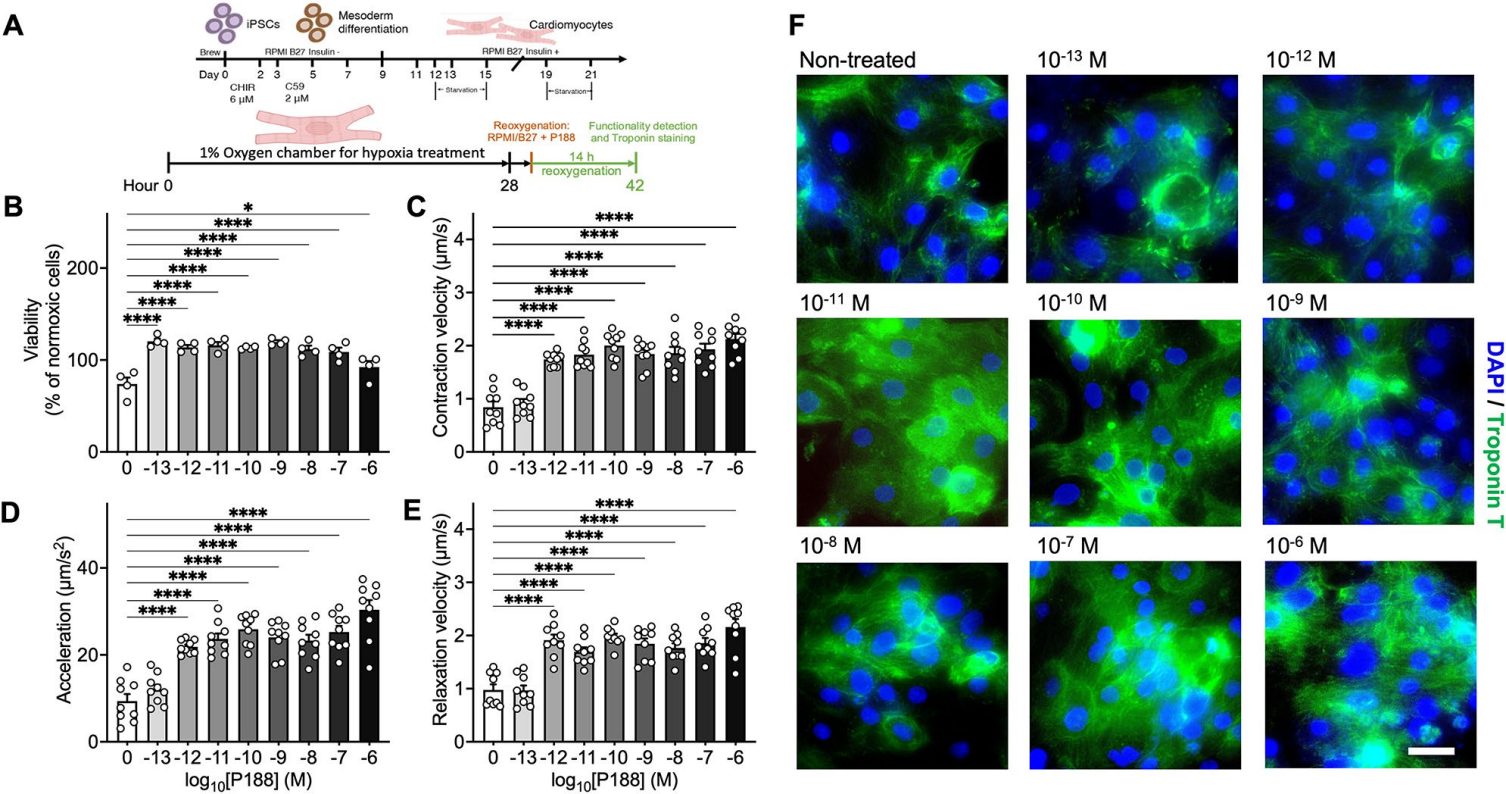
P188-mediated protection of iPSC-CMs under simulated H/R conditions. (**A**) Schematic diagram illustrating the differentiation process of iPSCs to iPSC-CMs, followed by hypoxic challenge and subsequent reoxygenation phase supplemented with various P188 concentrations. (**B**) Quantitative assessment of iPSC-CM viability across different P188 concentrations under simulated H/R conditions. The data emphasizes a marked increase in cell survival with increasing P188 concentrations. Cell viability values are normalized to normoxic control cells. (**C**–**E**) Quantitative representation showcasing the augmented functionalities of iPSC-CMs, including contraction rate (**C**), relaxation rate (**D**), and acceleration (**E**), upon P188 exposure under simulated H/R conditions. (**F**) Representative images depicting the distribution of troponin T in iPSC-CMs treated with varying P188 concentrations. Staining was shown as nuclei in blue and troponin in green; scale bar, 60 μm. Data are shown as mean ± SEM. Significance levels in comparison to the non-treated control group were determined using one-way ANOVA with Dunnett’s test: *P < 0.05, ****P < 0.0001.

iPSC-CMs were subjected to hypoxia by exposing them to 1% O_2_ for 28 h in a glucose-deprived medium to mimic hypoxic conditions. Following the hypoxia phase, iPSC-CMs were provided with a glucose-containing and oxygen-rich medium supplemented with varying concentrations of P188 to assess its protective effects (Fig. 1A). Remarkably, the protective effects of P188 on iPSC-CMs increased with higher concentrations, achieving nearly 90% cell survival (Fig. 1B), in contrast to the 50% survival in the untreated group. Moreover, P188 treatment significantly enhanced iPSC-CM function, including contraction rate, relaxation rate, and acceleration (Fig. 1C-E), suggesting its role in preserving and recovering iPSC-CM function under hypoxic conditions. The morphological evaluation of iPSC-CMs at different P188 concentrations (Fig. 1F) revealed that untreated cells exhibited cell shrinkage and an unhealthy morphology, while P188 at concentrations ranging from 1 µM to 100 fM effectively maintained healthy and intact cellular morphology.

#### iPSC-derived CM cell viability after hypoxia/reoxygenation experiments

**Fig. 2:**
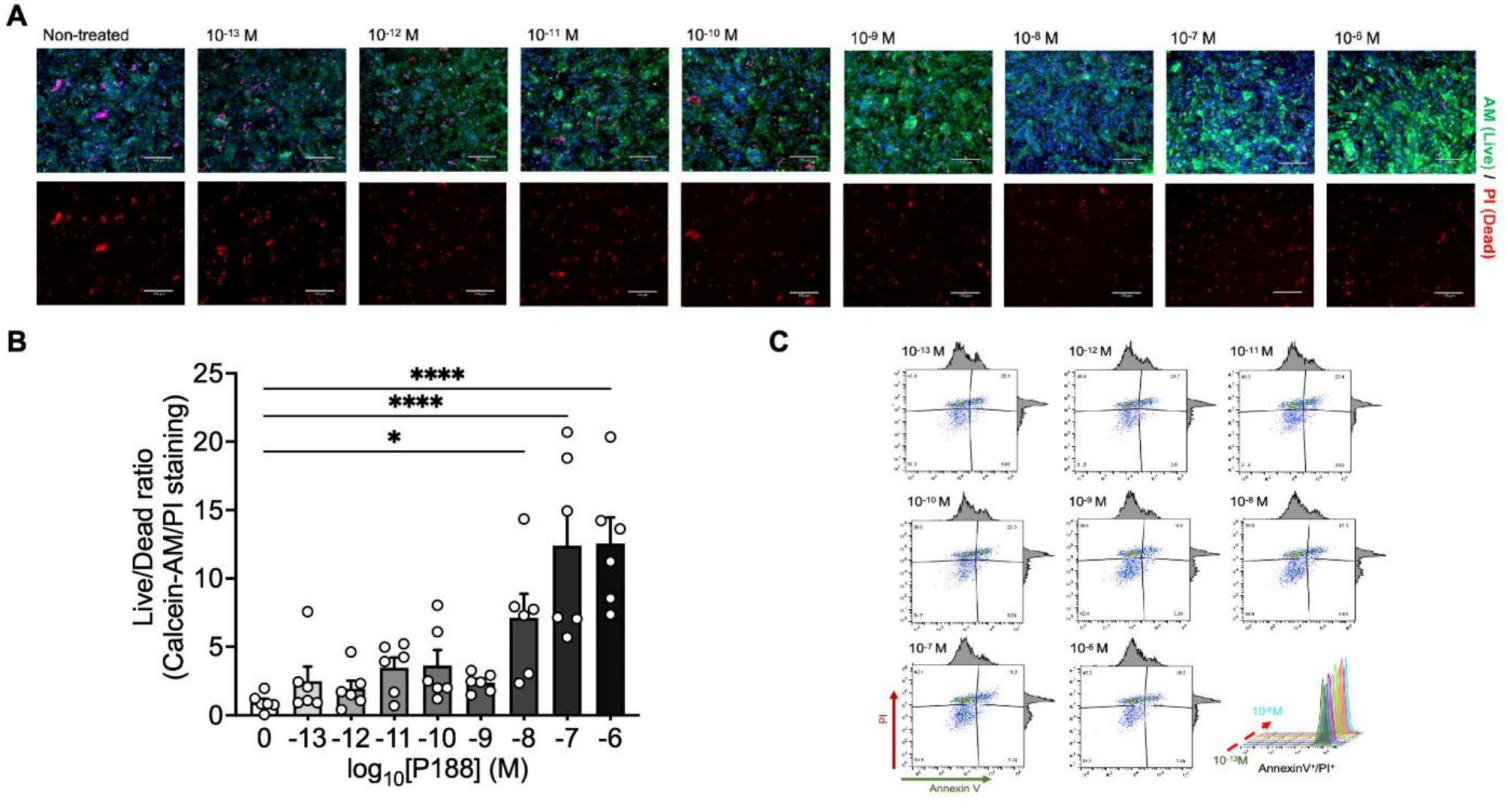
Cell death analysis and apoptosis detection in iPSC-CM. (**A**) Live/dead staining with Calcein-AM (green) and Propidium Iodide (PI, red) of iPSC-CMs after treatment with a concentration series of P188 ranging from 10^− 13^ M to 10^− 6^ M. Non-treated samples serve as controls. Scale bars, 170 μm. (**B**) Quantification of the live/dead cell ratio in iPSC-CMs following reoxygenation, with or without P188 treatment. (**C**) Flow cytometry analysis of Annexin V and PI staining for detection of apoptosis in iPSC-CMs. The stacked peaks represent the population of Annexin V-positive/PI-positive cells, indicating an increasing ratio of late apoptotic/necrotic cells in P188-treated iPSC-CMs. Data are shown as mean ± SEM. Significance levels in comparison to the non-treated control group were determined using one-way ANOVA with Dunnett’s test: *P < 0.05, ****P < 0.0001.

P188 restored intracellular adenosine triphosphate (ATP) levels after 14 h of reoxygenation as measured by the Cell titer glue assay (Fig. 1B). This effect was independent of the dose of P188 and might reflect the restored cellular metabolism in cells exposed to P188. In contrast, the cell live-dead staining indicated a dose-dependent protective effect of P188 against reoxygenation-induced injury in iPSC-CMs (Fig. 2A, B).

#### Annexin V positive iPSC-CM

To investigate the potential protective effect of P188 against programmed cell death, we quantified Annexin V-positive iPSC-derived CMs after 14 h of reoxygenation. Flow cytometric analysis revealed that P188 reduced the proportion of apoptotic cells from 3.7 to 2.0% (Fig. 2C). The lowest level of Annexin V-positive portion was observed in the group treated with 10^− 6^M.

Together, these findings underscore the potential of P188 as a promising therapeutic agent for preserving iPSC-CM viability and function during hypoxia-reoxygenation events.

#### iPSC-EC function after hypoxia/reoxygenation experiments

**Fig. 3:**
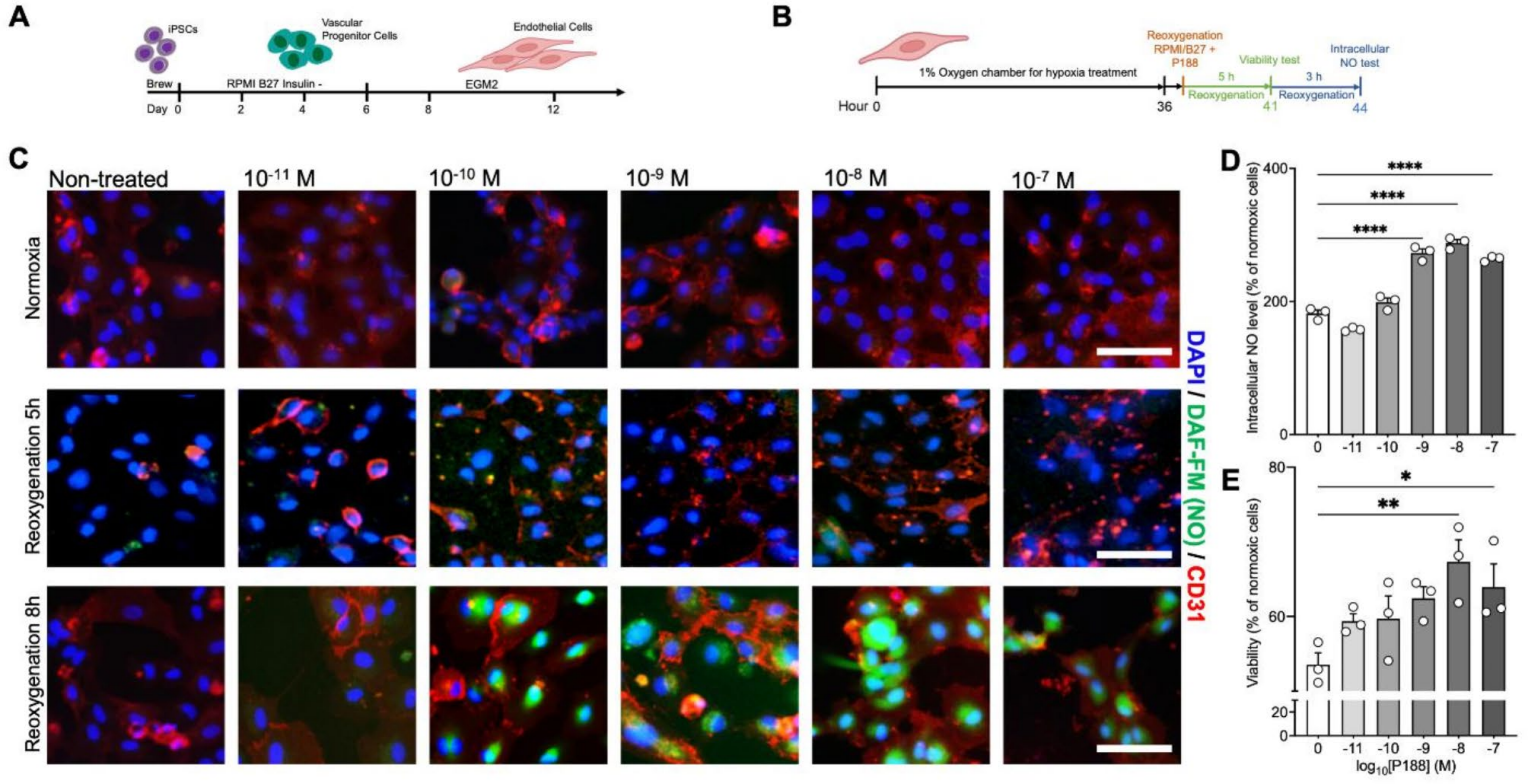
Effects of P188 on iPSC-ECs viability and intracellular NO production under simulated I/R conditions. (**A, B**) Schematic representation of the experimental timeline, detailing the differentiation protocol of iPSC-ECs (**A**) followed by hypoxic exposure and subsequent reoxygenation (**B**). (**C**) Representative images of intracellular NO staining, indicated by the DAF-FM dye. Positive intracellular NO production is depicted in green, nuclei are stained in blue, and the endothelial cell marker, CD31, is shown in red. The top row represents cells subjected to hypoxic treatment, while the bottom row shows corresponding cells under normal oxygen conditions, both treated with respective P188 concentrations; scale bars, 100 μm. (**D**) Quantification of intracellular NO production determined via DAF-FM staining; values normalized to normoxic control cells. (**E**) Quantification of cell viability post-reperfusion in iPSC-ECs treated with or without P188; values normalized to normoxic control cells. Data are shown as mean ± SEM. Significance levels in comparison to the non-treated control group were determined using one-way ANOVA with Dunnett’s test: *P < 0.05, **P < 0.01, ****P < 0.0001.

Moreover, P188 treatment also exhibited a notable effect on the intracellular production of NO. Quantitative analyses revealed that the rate of NO production in ECs treated with P188 increased by approximately 50% relative to the control group that did not receive P188 treatment (Fig. 3D). The concentration-dependent effects of P188 on ECs were particularly pronounced at a concentration of 10 nM, where the most substantial improvements in both endothelial cell protection and intracellular NO production were observed after 8 h of reoxygenation. Notably, the increase in NO production was not evident after 5 h of reoxygenation but became significant after 8 h ***(***Fig. 3C***)***. This temporal pattern suggests that the protective mechanisms activated by P188, which enhance NO production, may require a certain period before becoming fully operational within the cellular milieu. By enhancing EC survival and promoting NO release, P188 exhibits promising potential as a therapeutic agent to mitigate ischemic damage and improve cardiovascular outcomes.

### iPSC-derived EC cell viability after hypoxia/reoxygenation experiments

P188 treatment led to a significant increase in EC viability. When compared to the control group without P188 treatment, the percentage of viable ECs increased from 55% to nearly 70% (Fig. 3E).

### P188 effect on myocardial I/R injury in rat isolated hearts

#### Left ventricular function and infarct size

**Fig. 4:**
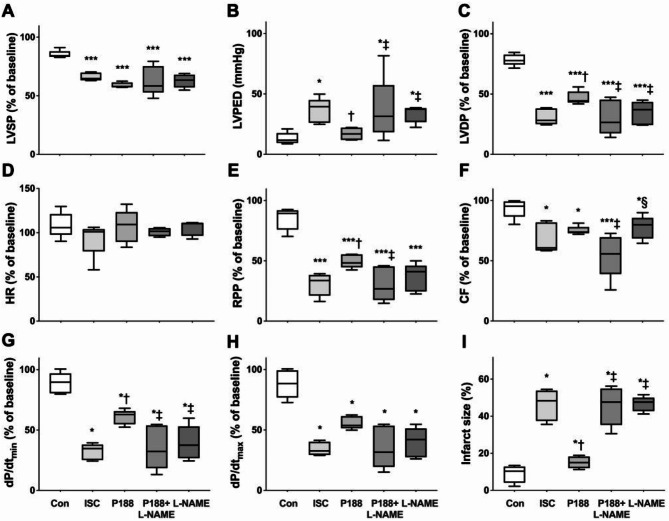
Effect of P188 on cardiac function and viability in rat isolated heart experiments. (**A**) Systolic (LVSP), (**B**) diastolic (LVEDP) and (**C**) developed left ventricular pressure (LVDP), (**D**) heart rate (HR), (**E**) rate pressure product (RPP), (**F**) coronary flow (CF), (**G**) dP/dt_min_ and (**H**) dP/dt_max_, as indices of relaxation and contractility, respectively, and (**I**) infarct size are shown at 120 min of reperfusion. P188 significantly improved cardiac function and viability when compared to other groups undergoing ischemia/reperfusion. Notably, inhibition of nitric oxide synthase by Nω-Nitro-L-arginine methyl ester hydrochloride (L-NAME) abolished all effects of P188. Since not all data were normally distributed and had equal variance, we display them as box plots with median and interquartile range. Tests were considered statistically significant at *P* < 0.05 (one symbol; *P* < 0.01 two symbols; *P* < 0.001 three symbols; *P* < 0.0001 four symbols): *vs CON/control, ^†^vs. ISC, ^‡^vs. P188, ^§^vs. P188 + L-NAME.

At the end of the 120 min reperfusion phase, all ischemic hearts displayed a significant decrease in LVSP, LVDP, RPP, dP/dt_min_, dP/dt_max_ and CF and a significant increase in infarct size (Fig. 4A, C, E, F, G, H, ***and Suppl. Table 2***) compared to non-ischemic control experiments. P188 administered on reperfusion led to a significant decrease in LVEDP ***(***Fig. 4B***)***, increase in LVDP, RPP, dP/dt_min_ and decrease in infarct size ***(***Fig. 4I, ***Suppl. Table 2***,*** Suppl. Figure 1)***. In P188 + L-NAME hearts, these improvements were abolished, while L-NAME itself had no significant effect by itself. There was no difference in HR among any of the groups ***(***Fig. 4D***)***.

#### NO measurements

**Fig. 5:**
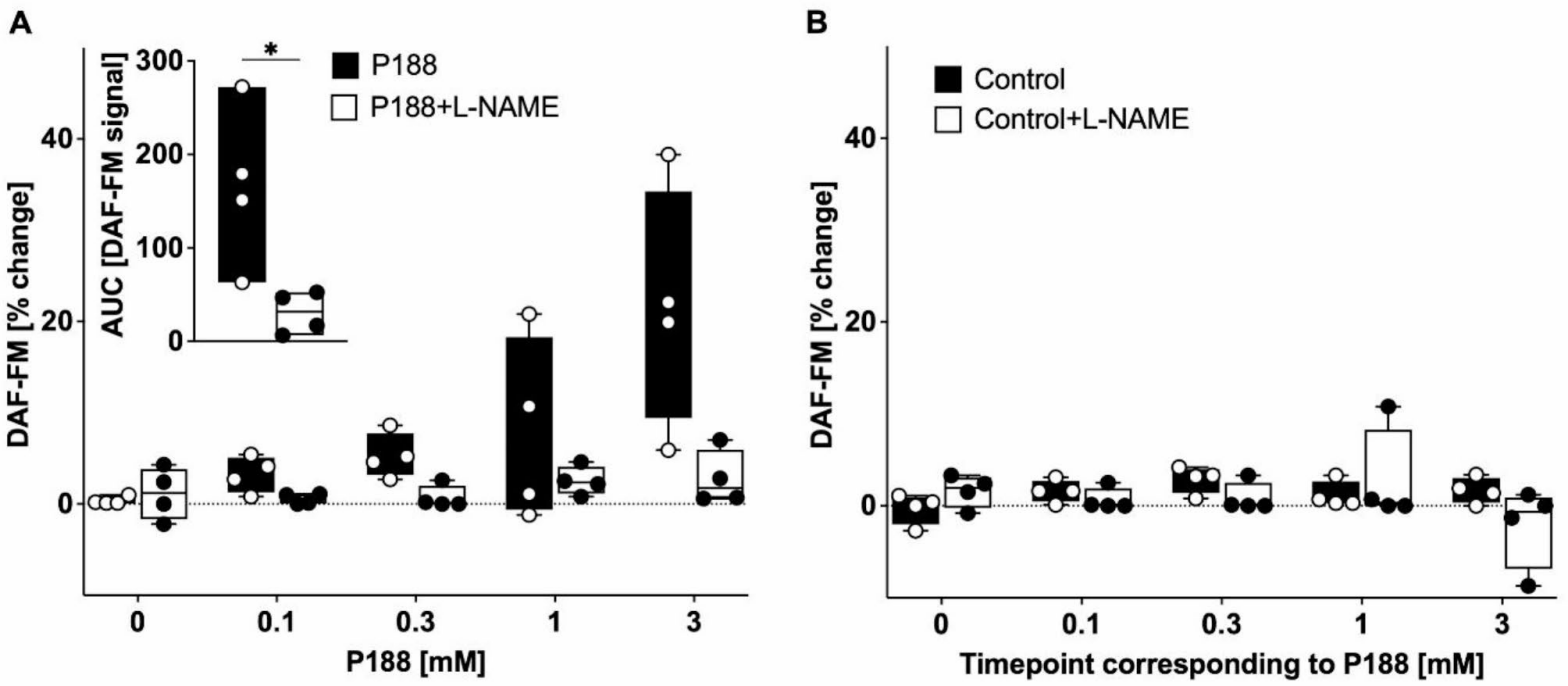
Nitric oxide measurement in rat isolated hearts at different P188 concentrations. (**A**) Fluorescence of the nitric oxide (NO)-sensitive dye 4-amino-5-methylamino-2′,7′-difluorofluorescein diacetate serving as an indicator of NO production revealed an increase after administration of P188 which was abolished by the nonspecific NO synthase inhibitor Nω-Nitro-l-arginine methyl ester hydrochloride (L-NAME). (A insert) In the absence of L-NAME, P188 increased the total amount of NO release, measured as the area under the curve (AUC) of the fluorescence signal; an effect which was abolished by L-NAME. (**B**) There was no increase in NO production in untreated control hearts. *P < 0.05 vs. P-188-NAME.

P188 dose-dependently increased NO fluorescence in the absence of L-NAME. This inducible effect was completely abolished by L-NAME ***(***Fig. 5A***)***. In contrast, there was no increase in NO fluorescence produced in untreated control hearts (Fig. 5B).

## Discussion

Our findings offer a novel insight into the multifaceted protective mechanisms of P188, highlighting its significant role beyond mere membrane stability. The ability of P188 to substantially increase NO production in ECs during reperfusion introduces a new dimension to its therapeutic potential. This increase in NO production, pivotal for vascular homeostasis and protection, suggests that P188’s benefits extend into modulating key signaling pathways involved in endothelial function and cardiovascular protection. P188-induced enhanced NO production may involve its amphipathic structure, which facilitates membrane stability and thereby potentially improves the functional status of membrane-bound enzymes such as eNOS. This hypothesis aligns with the observed delayed increase in NO production, suggesting a restoration of cellular functions because of preserved membrane integrity. These key results were validated in ex-vivo rat hearts, where P188 improved post-ischemia cardiac function in a NO-dependent manner. Moreover, P188 dose-dependently increased NO levels in non-ischemic beating hearts.

### Studies conducted in human induced pluripotent stem cell-derived CMs and ECs

We exposed both human iPSC-derived CMs and ECs to H/R stress and found that P188 dose-dependently protected both types of cells against reoxygenation injury. Human iPSC-derived CMs have been frequently used to study H/R injury^24,26–28^. Our findings align with our previous studies showing that P188 can provide protection against reoxygenation injury^29^ and that this protection depends on the dose of P188^30^. These studies suggested that P188, similar to other poloxamers, may stabilize cell membranes or improve cell viability under stress conditions^30^. The mechanism is thought to involve the surfactant’s ability to integrate into the lipid bilayer of cell membranes, thereby reducing membrane stress and preventing leakage of intracellular components^31,32^. Here, we utilized iPSC-CMs differentiated in regular RPMI/B27 medium, which made the cells relatively more resistant against hypoxic conditions compared to mature iPSC-CMs^22^, requiring a longer hypoxia duration compared to our previous study^33^. Nevertheless, we could demonstrate that P188 given during reoxygenation resulted in a 25% reduction of H/R injury. Uncontrolled intracellular Ca^2+^ levels can result in CM hypercontractility and cell death^34^. However, our previous experiments in CM revealed, that P188 did not target Ca^2+^ channels or exchangers when administered during reoxygenation; thus, the protective effect of P188 most likely stems from its ability to repair membrane disruptions, and thereby restore membrane integrity and cellular function^35^.

We detected only a modest increase in viability by P188. This might in part be caused by the fact that Annexin V positive cells were measured late (i.e., 13 h after reoxygenation) and emphasizes the acute effect of P188 on cell integrity upon reoxygenation.

Studies focusing on the examination of P188 in human tissue are still scarce, with only one prior publication conducted by Lin et al., which exclusively employed iPSC-CMs^36^. Remarkably, our investigation stands as the second study investigating the effects of P188 utilizing iPSCs, encompassing both iPSC-derived CMs and ECs. In contrast to the CM-only model employed by Lin et al., our bicellular model facilitates an enriched understanding of P188’s capabilities. Specifically, our research unveils the previously unexplored capacity of P188 to increase the secretion of NO in ECs, in addition to its recognized competence in membrane reparation evident by its effect on CM cell integrity^31,32,37^.

### Langendorff experiments

#### Ischemia/reperfusion studies

Our findings unequivocally reveal a noteworthy improvement in cardiac function across an extensive array of measured variables, accompanied by a considerable reduction in infarct size. The decrease observed in LVEDP among hearts subjected to P188 treatment primarily stems from enhanced relaxation during reperfusion. Similar investigations simulating myocardial infarction and congestive heart failure have consistently reported analogous outcomes, characterized by amplified LV end diastolic volume at reduced pressures, thereby yielding increased ejection fraction and cardiac function^29^. The state of constitutively overexpressing inducible NO synthase has previously demonstrated a comparable reduction in LVEDP upon reperfusion^38^. In our study, the observed improvement in LVEDP was entirely nullified by the administration of the non-specific NOS inhibitor L-NAME, indicating NO as key element in the protective mechanism exerted by P188. Furthermore, a strong cardioprotective effect has also been observed in an in-vivo porcine model when P188 was given immediately upon reperfusion following a prolonged coronary occlusion^39^. Given the statistical significance in I/R studies involving P188 and L-NAME, we are confident that P188, at least in part, is mediated by NO production.

### NO studies

The results of the I/R studies prompted further investigation into the dose-dependent effects of P188 on NO production in naïve non-ischemic hearts. The initial studies elicited results supporting the hypothesis that P188 directly increases the rate and quantity of NO production in healthy, non-ischemic hearts. Additionally, preliminary studies (not shown) involving microscopic analysis of major heart vessels, revealed that hearts treated with P188 demonstrated significantly higher amounts of NO being produced^40^. Our results show an increased absolute amount of NO produced in hearts treated with increasing concentrations of P188 which was abolished in the presence of L-NAME (Fig. 5A). Moreover, the potential of P188 to act as an oxygen radical scavenger could also augment the availability of NO, as demonstrated by previous studies indicating that the mitigation of oxygen radical burden during reoxygenation via endothelial superoxide dismutase overexpression results in elevated NO availability^41^. However, the impact of ROS on NO signaling is complex^42–44^. While moderate ROS levels can enhance NO bioavailability through redox-sensitive pathways such as PI3K/Akt signaling, excessive ROS reacts with NO to form peroxynitrite, a highly reactive nitrogen species that impairs endothelial function and reduces NO availability^45^. Importantly, activation of the PI3K/Akt pathway^46^ plays a crucial role, as Akt phosphorylation correlates with reduced infarct size and decreased mPTP formation during early reperfusion.

NO stabilizes calcium homeostasis and attenuates calcium overload in CMs^47,48^, activating key intracellular protein kinase (PK) pathways that inhibit mitochondrial permeability transition pore (mPTP) opening^49–51^, scavenging reactive oxygen species and suppressing oxidative damage, optimizing oxygen metabolism^41^ and energy production in CMs^52,53^.

It is known that during cardiac ischemia, the cellular membranes of cardiac ECs begin to degenerate following cellular swelling and extreme intracellular changes affecting multiple ion channels (for review see^54^). P188 has been shown to integrate into the damaged membranes to provide temporary protection and restore a physiological state until this lipid bilateral regenerates^32,55^. Membrane integrity is imperative for prevention of microembolism of endothelial microaggregates leading to a no-reflow phenomenon^56^. Our findings suggest that P188 assists in sealing breaches in the degenerating membrane and thereby suppress release and aggregation of denaturated endothelial membrane proteins^57^.

### Limitations

We had decided to recruit only male BN rats to avoid gender, hormonal cycle, body weight (female BN rats are about half the weight of males at the age we have used) and, thus, heart size as confounding factors in our study; this is a strength as much as it is a limitation to be considered. The treatment of hearts in ventricular fibrillation with lidocaine necessary for the continuation of the experiments but might have influenced the degree of measured cardioprotection by P188.

We know from previous ex-vivo experiments that myocardial oxidative stress is largely reduced by different cardioprotective measures such as ischemic or anesthetic preconditioning^58,59^ or hypothermia^60^. While our findings strongly suggest that P188 enhances cardioprotection through NO release from the endothelium, we acknowledge that a direct investigation of EC-CM crosstalk using co-culture systems would provide valuable mechanistic insights. Previous studies^61–64^ have demonstrated the critical nature of spatial and paracrine interactions between EC and CM in cardiac function and injury response, and preliminary data from our lab indeed point in this direction^65^. Future studies leveraging EC-CM co-culture platforms or engineered heart tissues would be instrumental in dissecting how P188 modulates EC-MC signaling, particularly regarding NO diffusion and intercellular communication under hypoxia/reoxygenation conditions.

Furthermore, the impact of reactive oxygen species (ROS) on NO signaling is complex. While moderate ROS levels can enhance NO bioavailability through redox-sensitive pathways such as PI3K/Akt signaling, excessive ROS reacts with NO to form peroxynitrite, a highly reactive nitrogen species that impairs endothelial function and reduces NO availability^45^. Furthermore, under oxidative stress, uncoupled endothelial NO synthase (eNOS) may shift from NO production to superoxide generation, further exacerbating endothelial dysfunction and I/R^66^ injury. Furthermore, under oxidative stress, uncoupled endothelial NO synthase (eNOS) may shift from NO production to superoxide generation, further exacerbating endothelial dysfunction and I/R injury^66^. This complex cascade of protective measures is initiated by flow-induced shear stress, which activates endothelial mechanosensors such as VEGFR2, PECAM-1, and integrins^67–71^, leading to an increase in NO production through phosphorylation^72,73^ and increased expression of endothelial NO synthase (eNOS)^74^.

Activation of eNOS^75^ and inducible NOS (iNOS)^38^, as well as overexpression of endothelial superoxide dismutase (ecSOD)^41^, has been previously implicated in increased NO levels during myocardial ischemia-reperfusion injury. While P188 primarily exerts its effects extracellularly, the potential induction of myocardial NOS or ecSOD in mediating protection against hypoxia-reoxygenation injury cannot be excluded. Therefore, the complex interactions between NO and ROS in the context of P188 administration will be object of future studies.

## Conclusion

Our approach of combining iPSCs with an ex-vivo animal model demonstrates that the amphiphilic copolymer P188 elicits cardioprotection against simulated I/R injury through coordinated membrane stabilization and an NO-dependent mechanism. P188 protected human iPSC-derived CMs and ECs from H/R damage while boosting endothelial NO production. In rat isolated hearts, P188 improved post-ischemia cardiac function and viability in a NO-dependent manner and increased NO levels dose-dependently. These findings substantiate the previously reported potential of P188 – and of newer di-block derivatives^76^ – as a novel therapy for I/R injury, warranting further evaluation and leveraging its dual capacity for membrane repair and NO potentiation during reperfusion^64,65^. Cocultures with a close spatial relation between CMs and ECs will further help in elucidating the interplay of these two cell types in P188-induced cardioprotection^64^.

## Materials and methods

All used materials and reagents are listed in supplement Table 1 **(Suppl. Table 1)**.

### Human induced pluripotent stem cells (iPSC)

The iPSC line was obtained from the Stanford Cardiovascular Institute Biobank (http://med.stanford.edu/scvibiobank.html*).* Patients were enrolled under the Stanford Institutional Review Board and Stem Cell Research Oversight Committee guidelines. Patients consent was retrieved and the iPSCs were generated from peripheral blood mononuclear cells (PBMCs) using Sendai virus (CytoTuneTM-iPS 2.0 Sendai Reprogramming Kit, Thermo Fisher Scientific) as previously described. Differentiation of iPSCs into iPSC-ECs^77,78^ and -CMs^79^ followed previously published protocols (Fig. 1A ***top panel***, Fig. 3A). The use of the lines was approved by the Administrative Panel on Human Subjects Research (IRB) under IRB #29,904 “Derivation of Human Induced Pluripotent Stem Cells (Biorepository)”.

### Differentiation of iPSC-CMs

iPSCs were next cultured to 85% cell confluence and then treated for 2 days with the GSK 3 inhibitor CHIR99021 (6 µM, Selleck Chemical) in RPMI medium plus B27 supplement without insulin medium to activate WNT signaling and induce mesodermal differentiation. On day 2, cells were placed on RPMI medium plus B27 supplement without insulin medium and CHIR99021(3 µM)^79,80^. On days 3–4, cells were treated with C59 (2 µM, Selleck Chemical) to inhibit the WNT pathway and induce cardiogenesis. On days 5–6, C59 was removed, and cells were cultured in RPMI medium plus B27 supplement without insulin medium. From day 7 onwards, cells were cultured in RPMI medium plus B27 supplement with insulin medium until cardiac contractions were observed. At this point, cells were cultured for 4 days in glucose-free RPMI medium plus B27 supplement (Gibco) with insulin medium to purify iPSC-CMs. Following purification, cells were cultured in RPMI medium plus B27 with insulin medium which regular media changes every other day (Fig. 1A, ***top panel***). When re-plating iPSC-CMs for downstream use, cells were dissociated with Trypsin/EDTA (0.25%, Life Technologies) into a single-cell suspension and seeded on Matrigel-coated plates with ROCK inhibitor and knockout serum replacement (1%, Gibco).

### Differentiation of iPSC-ECs

Briefly, iPSCs were dissociated using 0.5 mM EDTA/ phosphate-buffered saline (PBS, Innovative Cell Technologies) and plated as single cells in Stem cell brew (Miltenyi Biotec) supplemented with ROCK inhibitor Y-27,632 (Selleck Chemical) on Matrigel to a final density of 20,000–30,000 cells/cm^2^. iPSC monolayers were cultured to 85% cell confluency. iPSCs were then treated with the glycogen synthase kinase (GSK) 3 inhibitor CHIR99021 (6 µM, Selleck Chemical) for 2 days in RPMI medium plus B27 supplement without insulin (Gibco, CA). On day 2, cells were treated for 48 h with a lower dose of CHIR99021 (2 µM) in RPMI medium plus B27 supplement without insulin medium (Gibco). On day 4, cells were treated with the recombinant human fibroblast growth factor-2 (rhFGF-2, 10 ng/ml, PeproTech) and the vascular endothelial growth factor (VEGF, 20 ng/ml, R&D Systems) for EC expansion for 8 days. On day 12, cells were harvested with Trypsin/EDTA (0.25%, Life Technologies) and sorted using anti-CD144 MicroBeads (Thermo Fisher Scientific). The differentiation efficiency was calculated by the number of CD144-positive cells (Fig. 3A). iPSC-ECs were cultured in gelatin-coated (Sigma Aldrich) 6-well plates in EGM-2 medium (Lonza).

#### Hypoxia/reoxygenation experiments in iPSC-CMs and iPSC-ECs

iPSC-CMs were seeded at 30,000 cells per well on 384-well plates and cultured for 96 h prior to hypoxic/reoxygenation (H/R) treatment. iPSC-ECs were seeded on 24-well plates and cultured until reaching 95% confluence (2–3 days). To induce significant cell death (~ 50%), we followed previously published studies^22,81–83^ and exposed iPSC-CMs to 28 h of hypoxia (FiO_2_ = 0.01, measured with an FD-90 A-O_2_ oxygen meter) followed by 14 h of reoxygenation with varying P188 concentrations (Fig. 1A, ***lower panel***). iPSC-ECs were exposed to 36 h of hypoxia followed by 5–8 h of reoxygenation (Fig. 3B). The effects of P188 treatment on iPSC-EC viability, NO production, and CM functionalities were subsequently characterized.

### Cell viability assays in iPSC-CMs and iPSC-ECs

Following hypoxic treatment, iPSC-CMs and iPSC-ECs were reoxygenated in RPMI plus supplement and insulin medium containing different concentrations of P188 for 14 h and 5 h, respectively. After reoxygenation with P188, cell viability was assessed by adding an equal volume of Titer-Glo reagent (Promega) to the medium and incubating for 3 min at room temperature. Plates were preheated to 37 °C for 7 min before measuring luminescence on a Promega GloMax Multi plate reader. Relative luminescence units (RLU) were recorded to quantify cell viability.

#### Viability of iPSC-CM detection by flow cytometry

Viability was detected using the Annexin V-FITC/PI Apoptosis Detection Kit (Vazyme, A211) according to the manufacturer’s instructions. Briefly, cells (5 × 10^5^) were harvested, washed twice with PBS, and resuspended in 1X Binding Buffer. The cell suspension was incubated with 5 µl Annexin V-FITC and 5 µl PI Staining Solution for 10 min at room temperature in the dark. After incubation, 400 µl of 1X Binding Buffer was added, and the stained cells were analyzed within one hour on a table-top flow cytometry (BD C6 Accuri Flow Cytometer) using the 488 nm laser for excitation and the FL1 channel (FITC) and FL3 channel (Propidium Iodide [PI], Invitrogen) for detection. A total of 10,000 events were collected for each sample. Data analysis was performed using FlowJo software (version 10.8.1). The cells were classified into three subpopulations based on Annexin V-FITC and PI staining: viable cells (AnnexinV-FITC^-^/PI^-^), early apoptotic cells (AnnexinV-FITC^+^/PI^-^), and late apoptotic/necrotic cells (AnnexinV-FITC^+^/PI^+^).

### Measurement of iPSC-CM contractility

The contractility of iPSC-CMs was assessed after 14 h of reoxygenation using a cell motion imaging system (Sony SI8000, Sony Biotechnology). Videos of beating iPSC-CMs were recorded for 10 s at 75 frames per second (fps) with a resolution of 2048 × 2048 pixels using a 10× objective lens. CM contraction was analyzed using Sony SI8000 analysis software as previously described^84^.

### Measurement of nitric oxide production in iPSC-ECs

Intracellular NO production was assessed during reoxygenation by live-cell staining with the NO-sensitive fluorescent dye 4-Amino-5-Methylamino-2’,7’-Difluorofluorescein (DAF-FM) diacetate (Invitrogen). iPSC-ECs were stained with 1 µM DAF-FM diacetate for 10 min at 37 °C, in the last 10 min of reoxygenation at 7 h 50 min after reoxygenation commenced. Subsequently, Hoechst 33,342 (1 µg/mL, Thermo Scientific) was added to label nuclei. Imaging was performed on a Revolve microscope (Echo Laboratories) under 10× magnification. DAF-FM signals were detected in the GFP channel and Hoechst signals in the DAPI channel. DAF-FM fluorescence was also quantified using the GFP channel of a Cytation5 imaging system (BioTek, Agilent Technologies).

### Rat isolated heart experiments

#### Animals

All investigations abided by the Guide for the Care and Use of Laboratory Animals (Institute for Laboratory Animal Research, National Academy of Sciences, 8th edition, 2011), the ARRIVE guidelines^85^, and were previously approved by the Institutional Animal Care and Use Committees of Vanderbilt University Medical Center and the Tennessee Valley Healthcare System - Veterans Affairs Medical Center (both in Nashville, TN, USA; M1700168). Juvenile (8–10 weeks old, 160–250 g body weight) male Brown Norway (BN) rats were obtained from Charles River Laboratories International, Inc. The BN rat was chosen as it has been identified to be the rat strain most resistant against myocardial I/R injury, and produces higher level of endogenous NO^29,39^. A total of 40 BN rats were used to complete the studies.

#### Langendorff heart Preparation

Animals were anesthetized with intraperitoneal injection of 100 mg/kg of 100 mg/mL ketamine (Hospira) followed by intraperitoneal injection of 3,000 U/kg heparin (Fresenius Kabi). At this dose and timing, ketamine does not elicit or abrogate cardioprotection in a subsequent ex-vivo preparation^86^. After a negative response to a noxious stimulus 10 min later, animals were decapitated and hearts were excised via thoracotomy, following rapid cannulation of the aorta, distal to the aortic valve, and ligation of the inferior and superior venae cavae. The hearts were perfused retrograde with 4 °C cold oxygenated Krebs-Henseleit solution with the following composition (in mM): 148 Na^+^, 4.7 K^+^, 1.2 Mg^2+^, 1.6 Ca^2+^, 127 Cl^−^, 27.8 HCO_3_^−^, 1.2 H_2_PO_4_^−^, 1.2 SO_4_^2−^, 5.5 glucose, 2 pyruvate, 0.026 ethylene diamine tetraacetic acid, and 5 U/L insulin. The heart was promptly placed into the support system and perfused at 70 mmHg and 37 °C. The perfusate was equilibrated with 95% O_2_ and 5% CO_2_ to maintain a constant pH of 7.40 (carbon dioxide partial pressure pCO_2_ 40 mmHg; oxygen partial pressure pO_2_ ~ 570 mmHg). The perfusate was filtered using 5 μm pore size filters in-line. Left ventricular pressure (LVP) was measured isovolumetrically via a saline-filled latex balloon (Radnoti LLC, Monrovia, CA) inserted directly into the left ventricle by way of the left atrium. The initial volume of the balloon was titrated to achieve a diastolic LVP of 10 mmHg at baseline so that any consequent increase reflected diastolic contracture. LVP-derived data included systolic (LVSP), diastolic (LVEDP), and developed (systolic minus diastolic) LVP (LVDP), and its maximum and minimum first derivatives (dP/dt_max_ and dP/dt_min_) as indicators of ventricular contractility and relaxation, respectively. Spontaneous heart rate (HR) was monitored via electrocardiogram using bipolar electrodes placed in the right atrial and right ventricular walls. The rate pressure product (RPP) was calculated as developed LVP × HR to correct for HR-induced decreases in LVDP due to changes in sarcoplasmic reticulum calcium release. Changes in coronary flow were measured by an in-line ultrasonic flowmeter (T106X; Transonic Systems).

### Ischemia/reperfusion studies in rat isolated hearts

Langendorff prepared hearts were randomized to reperfusion with vehicle (control, *n* = 5), 1.0 mM P188 (*n* = 5), 100 µM of the nonspecific nitric oxide synthase inhibitor Nω-Nitro-L-arginine methyl ester hydrochloride (L-NAME [Sigma Aldrich], *n* = 5), or 1.0 mM P188 and 100 µM L-NAME (*n* = 5). 1.0 mM of the polymer was chosen following a series of preliminary I/R studies using 0.1 mM P188, 0.3 mM P188, 1.0 mM P188, or 3.0 mM P188. Additionally, our prior experiments in different in-vitro models of cardiac I/R have identified 1 mM as the optimal concentration to elicit cardioprotection^33,87^. Hearts were allowed to stabilize for 25 min. Following baseline readings, hearts were subjected to 30 min of global no-flow ischemia, immediately followed by 120 min of reperfusion with continuous monitoring of LVP, heart rate, and coronary flow, followed by tissue harvest and ventricular infarct size assessment^88–90^. If ventricular fibrillation occurred during reperfusion, a bolus of lidocaine (250 µg) was immediately injected into the aortic cannula to convert hearts back into sinus rhythm. All data were collected from hearts naturally in, or converted to, sinus rhythm; this allowed experiments to continue to the end and prevented unduly confounding the outcome by the energy depletion of ventricular fibrillation.

### Infarct size measurement

At the end of each experiment, hearts were removed from the system and weighed after the removal of both atria. Using a heart matrix, the ventricles were cut into 2-mm transverse sections. Heart slices were then incubated with 2,3,5-triphenyltetrazolium chloride (1%, TTC, Sigma Aldrich) in KH_2_PO_4_ buffer (100 mM, pH 7.4, 37 °C) for 10 min to stain viable tissue red by dehydrogenase enzymes present in viable cells, with infarcted areas remaining white^91^. Each heart slice was digitally imaged on a green background, and their infarcted areas were measured via planimetry using Image J (Ver 2.14.0, NIH, Bethesda, MD) software, its ColorThreshold plugin, and a custom-developed macro to ensure unbiased measurements^92^. Infarcted areas were averaged based on their weight to calculate the total ventricular infarction of each heart^91^. In this global ischemia model, the area-at-risk constitutes 100% in each heart. Representative images are shown in supplement Fig. 1 (***Suppl. Figure 1***).

#### Nitric oxide production studies in normoxic rat isolated hearts

Non-ischemic Langendorff-prepared hearts were randomized to perfusion with either vehicle only (control, *n* = 3), or P188 (*n* = 3). Hearts were allowed to stabilize for 25 min, then loaded with 10 µM of the NO-specific DAF-FM. P188 was administered in increasing concentrations (0 mM, 0.1 mM, 0.3 mM, 1.0 mM, and 3.0 mM) for 10 min; vehicle control experiments were conducted for the same duration but without P188 infusion. DAF-FM fluorescence was subsequently measured using a spectrophotometer via a bifurcated fiber optic probe in contact with the left ventricular epicardial wall. Fluorescence was excited at 495 nm and emitted at 515 nm^93^. This experimental procedure was repeated in the same hearts after the addition of 100 µM L-NAME to each concentration of P188 and control. Thereafter, 10 µM of the exogenous NO donor sodium nitroprusside was added to perfusate to ensure the fluorescent signal was not saturated (not shown). Since DAF fluorescence is irreversible, i.e. additive, fluorescence increases from before, not the absolute values, were used as a surrogate of NO release.

### Statistical analysis

All analog signals recorded throughout the studies were digitized (NI USB-6343, National Instruments Corporation) and recorded at 200 Hz (Labview, National Instruments) for later analysis. All data were tested for normal distribution and equal variance. If the data did not pass both tests, we analyzed them by non-parametric testing with either the Kruskal-Wallis or the Mann-Whitney test as appropriate. If they did pass both tests, data were compared by parametric one-way analysis of variance. The Student-Newman-Keul test was used for post-hoc testing among the groups. Since not all data were normally distributed and had equal variance, we display all of them as box plots with median and interquartile range for consistency, unless otherwise indicated. For cell-based assays we determined differences compared to the control group using Dunnett’s post hoc test; they are displayed as means ± standard error. Tests were considered statistically significant at *P* < 0.05 (one symbol; *P* < 0.01 two symbols; *P* < 0.001 three symbols; *P* < 0.0001 four symbols): *vs CON/control, † vs. ISC, ‡ vs. P188, § vs. P188 + L-NAME.

## Electronic supplementary material

Below is the link to the electronic supplementary material.

Supplementary Material 1

Supplementary Material 2

Supplementary Material 3

Supplementary Material 4

## Data Availability

The datasets used and/or analyzed during the current study are available from the corresponding author on reasonable request.

## Declarations

### ARRIVE guidelines

All data in this study are reported in accordance with ARRIVE guidelines (https://arriveguidelines.org).
