# Supplementary material for: Cardioprotection by poloxamer 188 is mediated through increased endothelial nitric oxide production
Source: Sci Rep. 2025 Apr 30;15:15170. doi: 10.1038/s41598-025-97079-z (PMC12043958; doi:10.1038/s41598-025-97079-z)
Supplement: Supplementary file 1 — Supplementary Material 1 [file 41598_2025_97079_MOESM1_ESM.docx]

# Supplement Table 1. Reagents and Equipment

| **Reagent / Equipment** | **Catalog Number / Model** | **Supplier** |
| --- | --- | --- |
| Annexin V-FITC/PI Apoptosis Detection Kit | A211 | Vazymebiotech |
| B27 Supplement (plus insulin) | A1486701 | Gibco |
| B27 Supplement (minus insulin) | A1895601 | Gibco |
| StemMACS™ iPS-Brew XF, human | 130-104-368 | Miltenyi Biotec |
| Calcein-AM | C3100MP | Invitrogen |
| CHIR99021, Free Base | C-6556 | LC Laboratories |
| DAF-FM diacetate | D23844 | Invitrogen |
| EGM™-2 Endothelial Cell Growth Medium-2 | CC-3162 | Lonza |
| Fibroblast Growth Factor-2 (rhFGF-2) | P09038 | PeproTech |
| Hoechst 33342 | H3570 | Invitrogen |
| KnockOut™ Serum Replacement | 10828010 | Gibco |
| Matrigel® Growth Factor Reduced Basement Matrix | 356231 | Corning |
| Nω-Nitro-L-arginine methyl ester hydrochloride | N5751 | Sigma Aldrich |
| P188 | K4894 | Sigma Aldrich |
| Propidium Iodide (PI) | P3566 | Invitrogen |
| ROCK Inhibitor Y-27632 | Y-5301 | LC Laboratories |
| RPMI 1640 Medium | 11875135 | Gibco |
| RPMI 1640 Medium (Glucose-free) | 11879020 | Gibco |
| Titer-Glo Luminescent Cell Viability Assay | G9241 | Promega |
| Trypsin-EDTA (0.25%) | 25200056 | ThermoFisher Scientific |
| 2,3,5-triphenyltetrazolium chloride | T8877 | Sigma Aldrich |
| UltraPure 0.5M EDTA | 15575-038 | Invitrogen |
| Wnt-C59 (C59) | S7037 | Selleck Chemical |
| **Equipment** | | |
| AC converter NI USB-6343 |  | National Instruments Corporation |
| Hypoxia Incubator Chamber | 27310 | STEMCELL Technologies |
| Cell Motion Imaging System | SI8000 | Sony Biotechnology |
| Microscope | ECHO-Revolve | ECHO |
| Flow Cytometer | Accuri C6 | BD Biosciences |
| Multi Plate Reader | GloMax | Promega |
| Oxygen Detector 0-30% | FD-90A-O2 | Forensics Detectors |
| Ultrasonic Flowmeter | T106X | Transonic Systems |
| **Software** | | |
| Image J | Ver. 2.14.0 | NIH |
| GraphPad Prism | Ver. 10 | Graphpad Software, LLC. |
| Labview | Ver. 2014 | National Instruments |
| R studio | Ver. 2023.06.2 | Posit Software |
| SI8000 | Ver. 1.03.0008 | Sony Biotechnology |
