# Supplementary figures and images for: Cardioprotection by poloxamer 188 is mediated through increased endothelial nitric oxide production
Source: Sci Rep. 2025 Apr 30;15:15170. doi: 10.1038/s41598-025-97079-z (PMC12043958; doi:10.1038/s41598-025-97079-z)

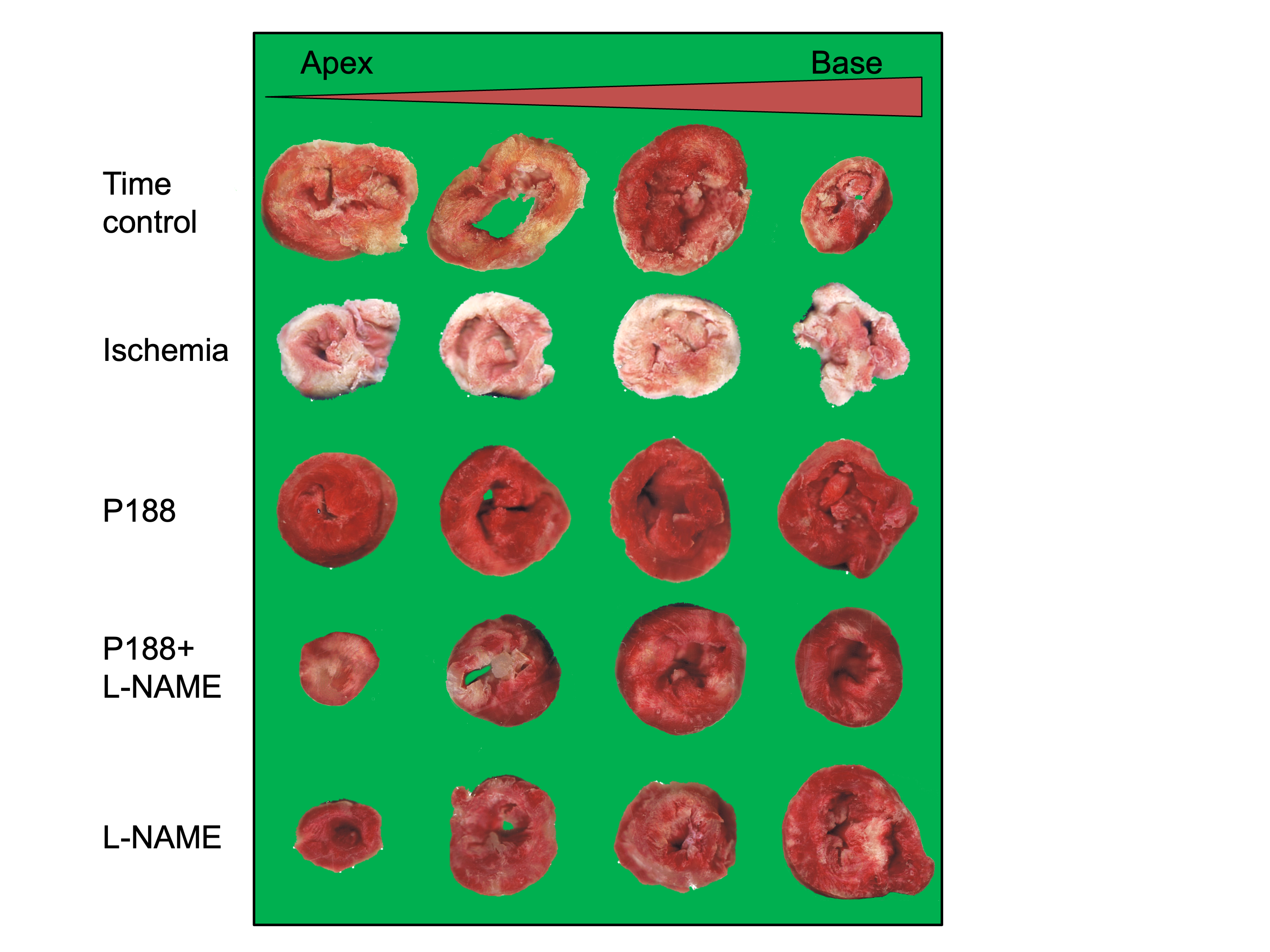

Supplement: Supplementary file 2 — Supplementary Material 2 [file 41598_2025_97079_MOESM2_ESM.tiff]
