# Supplementary material for: Cardioprotection by poloxamer 188 is mediated through increased endothelial nitric oxide production
Source: Sci Rep. 2025 Apr 30;15:15170. doi: 10.1038/s41598-025-97079-z (PMC12043958; doi:10.1038/s41598-025-97079-z)
Supplement: Supplementary file 3 — Supplementary Material 3 [file 41598_2025_97079_MOESM3_ESM.docx]

| **Supplement Table 2: Original Functional Langendorff and Infarct Size Data** | | | | | | | |
| --- | --- | --- | --- | --- | --- | --- | --- |
| **Group** | **Baseline** | **120 min Reperfusion** | **Group** | **Baseline** | **120 min Reperfusion** | | |
| **LVSP (mmHg)** | | | **LVEDP (mmHg)** | | | | |
| Con | 80.3 (57.8; 95.3) | 66.7 (48.3; 84.0) | Con | 10.0 (9.8; 10.4) | 11.7 (9.8; 15.9) | | |
| ISC | 87.6 (74.4; 114.0) | 51.4 (44.5; 74.8) | ISC | 9.8 (8.1; 10.9) | 39.4 (26.7; 42.5) | | |
| P188 | 86.8 (74.9; 129.8) | 51.2 (42.4; 70.8) | P188 | 9.9 (9.6; 10.0) | 16.9 (11.9; 21.9) | | |
| P188&L-NAME | 83.5 (72.6; 105.8) | 49.1 (40.2; 68.9) | P188&L-NAME | 9.8 (8.8; 10.5) | 31.5 (21.6; 45.1) | | |
| L-NAME | 112.4 (96.9; 115.2) | 65.8 (60.5; 69.3) | L-NAME | 9.8 (8.6; 10.1) | 37.3 (28.9; 38.0) | | |
| **dP/dt_max_  (mmHg s^-1^)** | | | **dP/dt_min_ (-mmHg s^-1^)** | | | | |
| Con | 1179 (735; 2194) | 955 (683; 2100) | Con | 967 (1180; 602) | 815 (1080; 545) | | |
| ISC | 1538 (1148; 2919) | 637 (334; 1004) | ISC | 1315 (1805; 961) | 455 (540; 244) | | |
| P188 | 1620 (1335; 2547) | 870 (731; 1352) | P188 | 911 (1834; 788) | 577 (986; 507) | | |
| P188&L-NAME | 1811 (1757; 2615) | 728 (509; 885) | P188&L-NAME | 1078 (1639; 907) | 456 (479; 294) | | |
| L-NAME | 2689 (2464; 3033) | 1133 (759; 1323) | L-NAME | 1701 (1789; 1368) | 649 (743; 444) | | |
| **HR (bpm)** | | | **RPP (10^4^ mmHg bpm)** | | | | |
| Con | 268 (217; 272) | 284 (208; 298) | Con | 18.6 (11.0; 22.2) | 12.3 (9.8; 9.1) | | |
| ISC | 267 (253; 307) | 261 (216; 308) | ISC | 23.0 (17.0; 28.6) | 6.2 (2.6; 7.8) | | |
| P188 | 222 (218 ;284) | 255 (244; 270) | P188 | 17.1 (14.2; 34.1) | 8.0 (6.9; 13.4) | | |
| P188&L-NAME | 261 (248; 290) | 273 (244; 297) | P188&L-NAME | 18.9 (18.3; 22.6) | 4.0 (3.7; 7.7) | | |
| L-NAME | 266 (261; 281) | 293 (277; 296) | L-NAME | 28.3(22.8; 29.1) | 9.1 (6.4; 12.3) | | |
| **LVDP (mmHg)** | | | **CF (ml min^-1^ g^-1^)** | | | | |
| Con | 68.8 (48.5; 85.3) | 54.9 (36.3; 66.1) | Con | 11.1 (6.1; 17.4) | 11.1 (5.8; 14.8) | | |
| ISC | 79.5 (63.0; 104.7) | 19.1 (13.3; 27.5) | ISC | 12.8 (8.8; 13.7) | 7.9 (6.3; 9.4) | | |
| P188 | 77.2 (64.8; 120.0) | 31.3 (27.7; 51.1) | P188 | 7.3 (7.0; 9.4) | 5.5 (5.4; 6.9) | | |
| P188&L-NAME | 72.0 (63.8; 95.7) | 17.7 (14.7; 23.6) | P188&L-NAME | 11.3 (10.2; 12.5) | 5.8 (3.8; 8.8) | | |
| L-NAME | 102.5 (87.3; 105.2) | 35.4 (22.1; 41.1) | L-NAME | 11.4 (9.9; 13.1) | 8.2 (7.5; 11.4) | | |
| **IS (%)** | | |  |  |  |  |  |
| Con |  | 11.1 (7.8; 29.4) |  |  |  |  |  |
| ISC |  | 48.3 (38.4; 53.5) |  |  |  |  |  |
| P188 |  | 15.0 (12.4; 17.9) |  |  |  |  |  |
| P188&L-NAME |  | 47.6 (37.6; 54.2) |  |  |  |  |  |
| L-NAME |  | 47.6 (43.5; 49.2) |  |  |  |  |  |
| **Supplement Table 2** shows summary of original data of P188 with or without Nω-Nitro-L-arginine methyl ester hydrochloride (L-NAME) vs LNAME alone in comparison to non-ischemic time control and ischemic control experiments at baseline and 120 min reperfusion in rat isolated (Langendorff) hearts. These are the basis for the % baseline data reported in Fig 4. Measured and calculated variables are: systolic (LVSP), diastolic (LVEDP) and developed left ventricular pressure (LVDP), heart rate (HR), rate pressure product (RPP), coronary flow (CF), dP/dt_min_ and dP/dt_max_, as indices of relaxation and contractility, respectively, and infarct size (IS, only at end of reperfusion). Analogous to Fig 4, we display data as median and interquartile range. bpm = beats per minute. | | | | | |  |  |
