# Supplementary material for: Cardioprotection by poloxamer 188 is mediated through increased endothelial nitric oxide production
Source: Sci Rep. 2025 Apr 30;15:15170. doi: 10.1038/s41598-025-97079-z (PMC12043958; doi:10.1038/s41598-025-97079-z)
Supplement: Supplementary file 4 — Supplementary Material 4 [file 41598_2025_97079_MOESM4_ESM.docx]

# **Legends to the figures and tables**

**Supplement Fig. 1** *Examples of 2,3,5-triphenyltetrazolium chloride-stained rat hearts after ischemia/ reoxygenation experiments.* Representative images of heart cross sections of all treatment groups. Time Control: hearts perfused for 175 min with Krebs-Henseleit perfusion buffer; Ischemia: hearts exposed to 30 min of global no-flow ischemia and 120 min of reperfusion; P188: hearts received 1.0 mM P188 during reperfusion. P188+L-NAME: hearts received 1.0 mM P188 and 10 µM of the nonspecific nitric oxide synthase inhibitor Nω-Nitro-L-arginine methyl ester hydrochloride (L-NAME); L-NAME: heart perfused with L-NAME during reperfusion. Heart sections are presented from apex (left) to base (right).

**Supplement Table 1** *Reagents and Equipment.* List of all used reagents and equipment.

**Supplement Table 2** *Original Functional Langendorff and Infarct Size Data.* Table shows summary of original data of P188 with or without Nω-Nitro-L-arginine methyl ester hydrochloride (L-NAME) vs L-NAME alone in comparison to non-ischemic time control and ischemic control experiments at baseline and 120 min reperfusion in rat isolated (Langendorff) hearts. These are the basis for the % baseline data reported in Fig 4. Measured and calculated variables are systolic (LVSP), diastolic (LVEDP) and developed left ventricular pressure (LVDP), heart rate (HR), rate pressure product (RPP), coronary flow (CF), dP/dt_min_ and dP/dt_max_, as indices of relaxation and contractility, respectively, and infarct size (IS, only at end of reperfusion). Analogous to Fig 4, we display data as median and interquartile range. bpm = beats per minute.
